# Case report: Reversible splenial lesion syndrome caused by diquat poisoning

**DOI:** 10.3389/fneur.2023.1178272

**Published:** 2023-08-17

**Authors:** Ping Dai, Jin Sun, Zhongkai Yu, Tongyue Zhang, Zixin Wen, Tianzi Jian, Lanlan Guo, Aerbusili Genjiafu, Baotian Kan, Binbin Zhang, Xiangdong Jian

**Affiliations:** ^1^Department of Poisoning and Occupational Diseases, Emergency Medicine, Qilu Hospital, Cheeloo College of Medicine, Shandong University, Jinan, Shandong, China; ^2^Emergency Department, Affiliated the Jianhu Clinical Medical College of Yangzhou University, Yancheng, China; ^3^Department of Emergency, Liaocheng People's Hospital, Liaocheng, Shandong, China; ^4^School of Nursing and Rehabilitation, Cheeloo College of Medicine, Shandong University, Jinan, Shandong, China; ^5^Nursing Theory and Practice Innovation Research Center of Shandong University, Jinan, Shandong, China; ^6^Department of Occupational and Environmental Health, School of Public Health, Cheeloo College of Medicine, Shandong University, Jinan, Shandong, China; ^7^Department of Geriatric Medicine and Department of Nursing, Qilu Hospital, Nursing Theory Innovation and Research Center of Shandong University, Cheeloo College of Medicine, Shandong University, Jinan, Shandong, China; ^8^Department of Nursing, Qilu Hospital of Shandong University Dezhou Hospital, Nursing Theory Innovation and Research Center of Shandong University, Cheeloo College of Medicine, Shandong University, Jinan, Shandong, China

**Keywords:** diquat, acute kidney injury, toxic encephalopathy, reversible splenial lesion syndrome, MRI

## Abstract

Diquat (DQ), chemically known as 1,1 ‘-ethylene-2,2’ -bipyridine, is a non-selective herbicide for leaf removal and drying. It has toxic effects on central nervous system cells, and toxic neurological lesions include axonal degeneration and pontine myelolysis. At the same time, DQ can also affect the activity of dopaminergic nerve cells through oxidative stress, causing degeneration and reducing dopamine uptake. With the increasing application of DQ in agricultural production, the clinical reports of neurotoxicity caused by acute DQ poisoning are also increasing. At present, DQ rapid-phase-related toxic encephalopathy mainly involves the pons, midbrain, basal ganglia, thalamus and other brain regions. However, this case is unusual in that the lesion mainly involved the splenium of the corpus callosum. It is also the first time to be reported.

## Introduction

Reversible splenial lesion syndrome(RESLES) is a clinical imaging syndrome. It was proposed by Garcia-Monco et al. based on imaging features (1). Imaging examination revealed that the characteristic change of this disease was a reversible lesion in the splenium of the corpus callosum. RESLES and mild encephalitis with a reversible splenial lesion of the corpus callosum (MERS) are different terms for the same clinical disease. There are a wide range of causes for RESLES. But there are few reports of pesticide poisoning causing RESLES so far. Diquat has not been reported in the literature to cause RESLES.

## Case description

**Figure 1:**
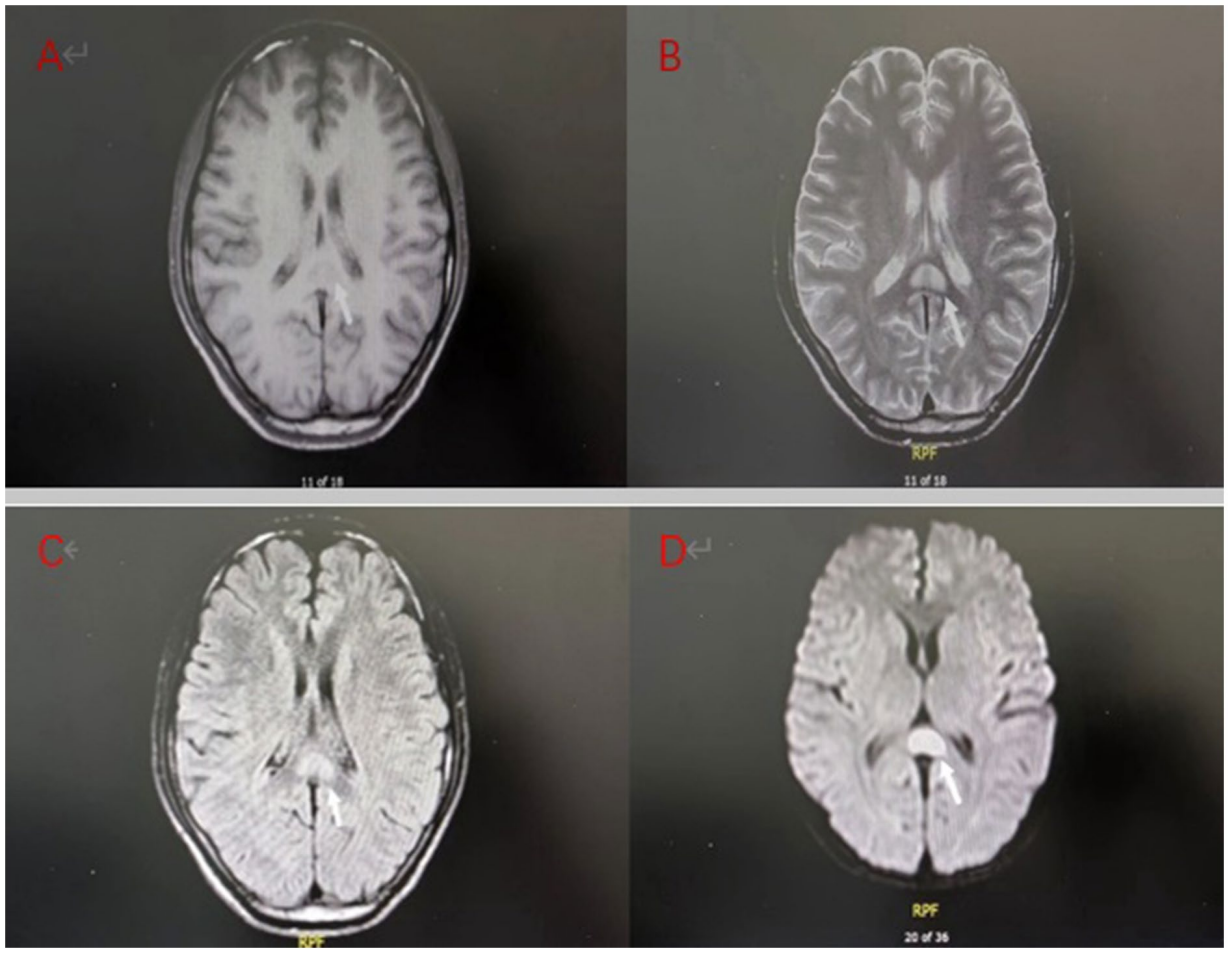
Brain MRI on admission of the patient: **(A)** T1 weighted imaging (T1WI) showed low signal intensity; **(B)** T2 weighted imaging (T2WI) showed high signal intensity; **(C)** Fluid attenuated inversion recovery (FLAIR) showed a high signal; **(D)** Diffusion weighted imaging (DWI) showed more pronounced hyperintensity than T2WI.

**Figure 2:**
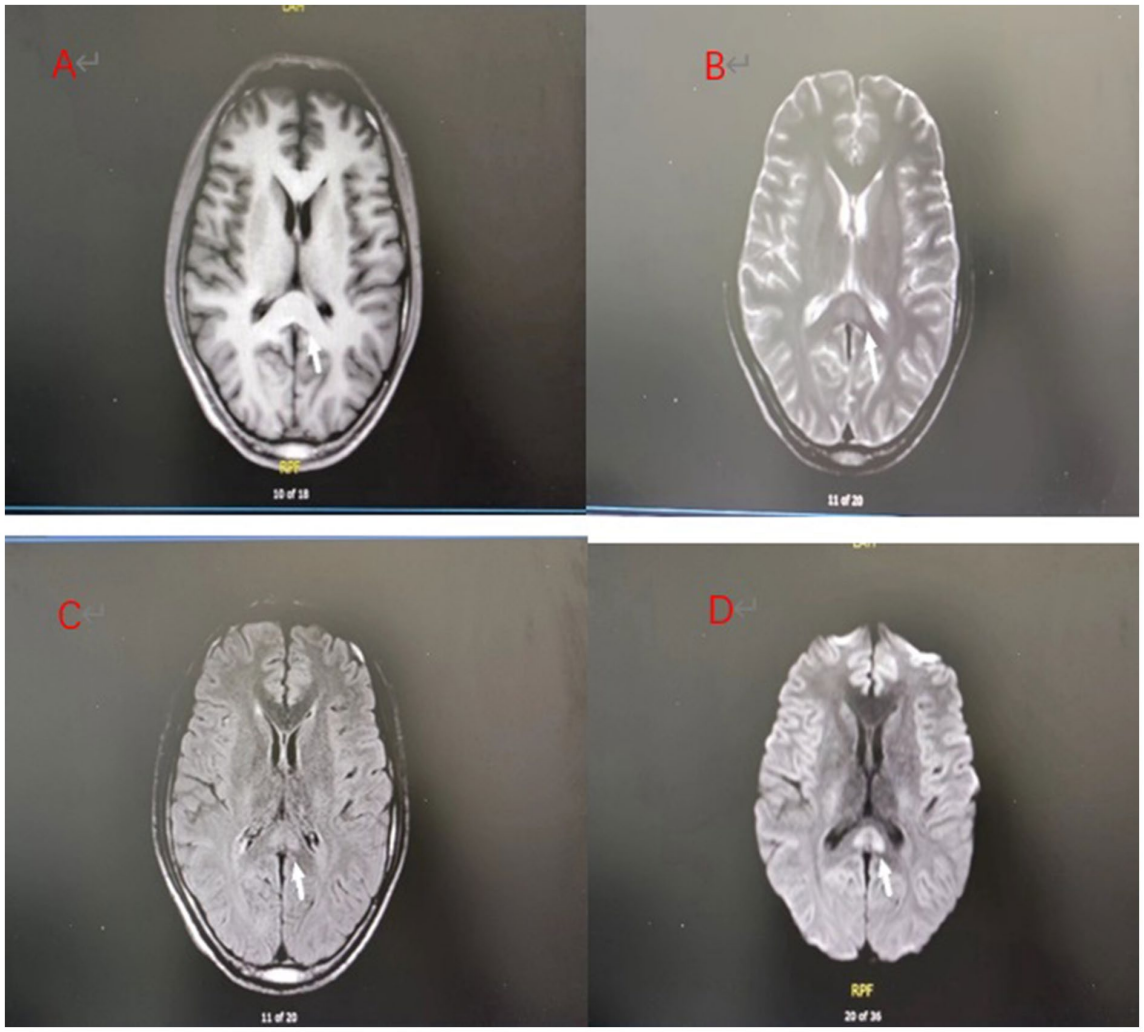
**(A–D)** Day 7 brain MRI:The splenial lesion of the corpus callosum had improved and might be considered as RESLES.

**Figure 3:**
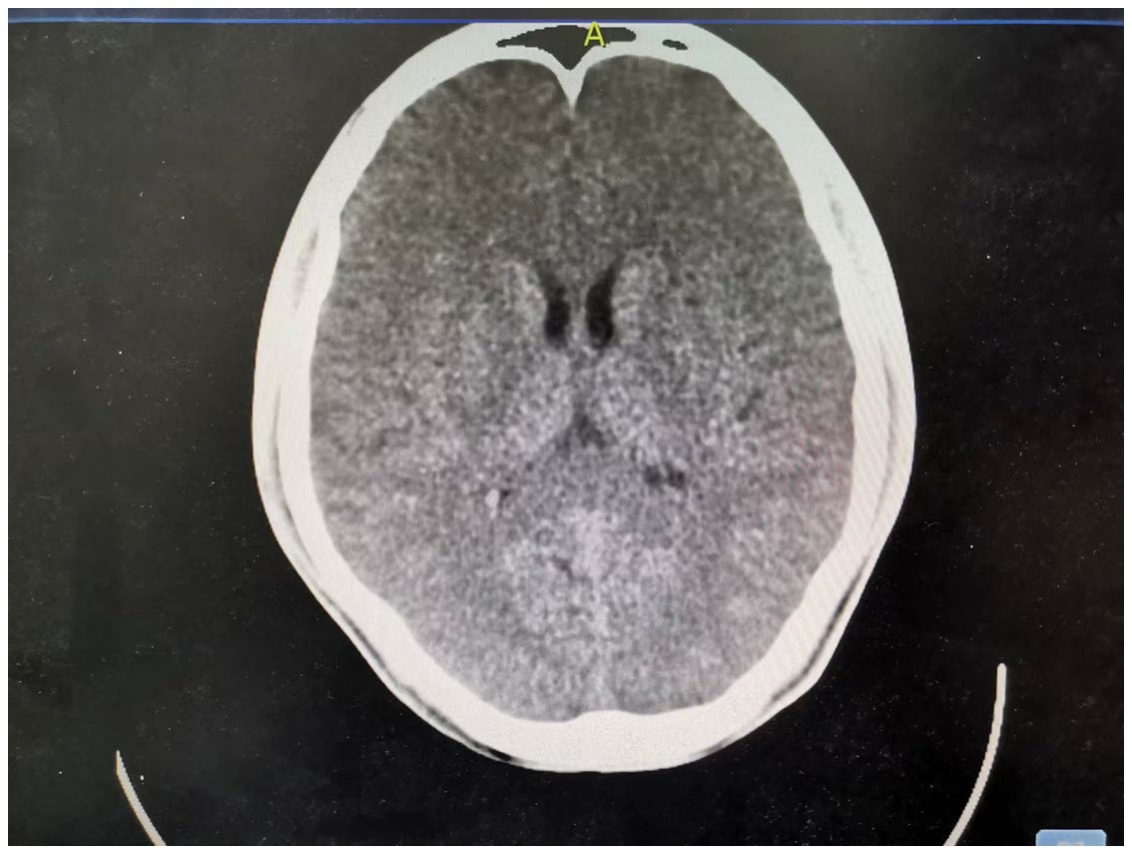
Day 14 brain CT: The splenial lesion of the corpus callosum was no longer visible on computed tomography scan.

A 25-year-old male patient was admitted to a local hospital with oral diquat 9 days ago. There, he was treated with gastric lavage, hemoperfusion, organ protection, and nutritional support. The renal function gradually deteriorated during the treatment, so he was transferred to our hospital on October 26. On admission, the patient was conscious and had a normal urine output. His vital signs were: body temperature 36.8°C, heart rate 87 beats /min, respiration 18 beats /min, blood pressure 126/72 mmHg, and physical examination was normal except for mild oedema of the lower extremities. Blood results were as follows: D-dimer at admission: 2.36 μg/mL (normal value, <0.5ug/ml), blood urea nitrogen (BUN):8.6 mmol/L (normal value, 3.2–7.1 mmol/L), creatinine (Cr):319 umol/L (normal value, 62–115 umol/L)；Other laboratory test results are shown in Table 1. The magnetic resonance imaging (MRI) examination of the head suggested that the lesions of the splenium of the corpus callosum, combined with the medical history, could be consistent with the MR manifestations of toxic encephalopathy (Figure 1). The patient was given betamethasone (8 mg, once a day), torasemide (20 mg, twice a day), alanylglutamine (10 g, once a day), lansoprazole (30 mg, twice a day), reduced glutathione (1.8 gram once a day) and nutritional support. On the 7th day of treatment, brain MRI showed that the abnormal signal of the splenium of the corpus callosum might be considered as RESLES (Figure 2). At this time blood tests showed: white blood cell count:13.75×10^9^/L(normal value, 3.5–9.5 ×10^9^/L), neutrophils:82.50%; d-dimer: 1.99 μ g/mL; BUN:22.20 mmol/L, Cr:203 umol/L. The patient’s renal function improved and lansoprazole, torasemide, betamethasone were discontinued. On day 10, his renal function had improved, and he was discharged from the hospital. On day 14, the splenial lesion of the corpus callosum is no longer visible on computed tomography scan (Figure 3). The patient’s laboratory test results are shown in Table 1.

**Table 1:** Biochemical blood test results.

Biochemical blood indicators	Our department				
	Normal values	10–26 18:33 (Day 1)	10–28 08:45 (Day 3)	11–01 07:51 (Day 7)	11–08 09:18 (Day 14)
WBC (×10^9^ /L)	3.5–9.5	6.66	16.58	13.75	19.43
NEU (%)	40–75	64.50	91.00	84.50	80.50
RBC (×10^12^ /L)	4.3–5.8	3.90	3.68	3.98	4.05
HGB (g/L)	130–175	124.0	118.0	126.0	125.0
PLT (×10^9^ /L)	125–350	193	235	299	218
ALT (U/L)	21–72	58	39	47	85
AST (U/L)	17–59	61	25	27	32
DBIL (μmol/L)	0–5	0	2.1	3.1	4.0
IBIL (μmol/L)	0–19	11	1.4	3.2	8.4
CK (U/L)	55–170	143	133	27	41
BUN (mmol/L)	3.2–7.1	8.6	16.20	22.20	10.50
Cr (μmol/L)	62–115	319	318	203	119

ALT, alanine transaminase; AST, aspartate aminotransferase; BUN, blood urea nitrogen; CK, creatine kinase; Cr, creatinine; DBIL; direct bilirubin; HGB, hemoglobin; IBIL; indirect bilirubin; NEU, neutrophils; PLT, platelets; RBC, red blood cells; WBC, white blood cells.

## Discussion

The characteristic findings of RESLES on brain MRI include lesions that are mostly confined to the splenium of the corpus callosum with clear boundaries; a few can involve the white matter area outside the splenium of the corpus callosum, without obvious oedema and mass effect around the lesions. T1-weighted imaging (T1WI) shows equal or slightly low signal intensity, T2-weighted imaging (T2WI) shows slightly high signal intensity, diffusion weighted imaging (DWI) shows more obvious high signal intensity than T2WI and apparent diffusion coefficient(ADC) shows low values, and most lesions disappear within 2 weeks. They can be divided into three categories: (a) small round or oval lesions at the midline of the splenium of the corpus callosum; (b) extention laterally through the fibers of the corpus callosum, showing the ‘boomerang sign’; (c) extension from the splenium of the corpus callosum to the anterior part of the corpus callosum and the deep white matter (2). The etiology is complex and diverse. In addition to seizures and withdrawal of antiepileptic drugs (3–5), the use of drugs, such as metronidazole (6), olanzapine (7), glucocorticoids (8), cisplatin, carboplatin, 5-fluorouracil and other antitumour drugs (1), and anorexic sympathetic weight loss drugs (1) can also cause RESLES. Other causes include influenza A (9), rotavirus infection (10), *streptococcus pneumoniae* (11), meningococcal meningitis (12), metabolic conditions such as hypoglycemia or hypernatremia (13, 14), glycophosate poisoning (15), and other diseases. These include vitamin B12 deficiency (16), malnutrition (17), high plateau brain oedema (18), systemic lupus erythematosus (SLE) (19), Kawasaki disease (20), anti-volt-gated potassium channel (VGKC) autoantibody syndrome (21), malignant tumours (14), cerebral venous sinus thrombosis (22), preeclampsia (23), cranial brain trauma (24) and renal failure (8).

The clinical manifestations of RESLES patients are diverse and non-specific. Disturbance of consciousness and headache are common symptoms. It can also manifest as fever, dizziness, vomiting, disturbance of consciousness, convulsions, delirium, blurred vision and paraesthesia. (25). Pathogenesis is unclear; the characteristics of the focal reversible change suggest that the pathogenesis is transient damage of the blood–brain barrier, reversible demyelination, oedema, cytotoxic oedema of myelin sheath, and arginine vasopressin (AVP) release. It may also be associated with short inflammatory reaction, elevated inflammatory cytokines and genetic factors (8, 26). Due to the complex etiology, RESLES may be the result of multiple mechanisms.

After admission, the patient’s brain MRI showed abnormal signal at the splenium of the corpus callosum.Splenic infarction of the corpus callosum was excluded because it is relatively rare for young patients to have splenic infarction (27), and there were no suggestive symptoms such as headache, dizziness or visual impairment. Apart from renal function, routine blood examination was normal, and neurological physical examination was negative. Therefore, no specific drugs for encephalitis or encephalopathy were used in the treatment process, and only 8 mg of betamethasone was given. After reexamination, abnormal signals were still found in the splenium of corpus callosum on brain MRI, but the range of high signal intensity on DWI was reduced compared with that on admission, and the signal intensity was also weakened; this was considered to be RESLES. Combined with the medical history, it was considered that it might have been caused by diquat poisoning. However, it could not be ruled out that it was caused by renal insufficiency and inflammatory reaction after diquat poisoning. It might also have been related to the use of glucocorticoids in the treatment of the diquat poisoning.

## Conclusion

Here we report for the first time a case of RESLES associated with diquat. Although the specific mechanism is unknown, based on the history and symptom analysis, we believe that it is related to diquat poisoning. RESLES has no specific clinical manifestations, mild symptoms and a good prognosis, but no neurological sequelae.

## Data availability statement

The original contributions presented in the study are included in the article/supplementary material, further inquiries can be directed to the corresponding authors.

## Ethics statement

Written informed consent was obtained from the individual(s) for the publication of any potentially identifiable images or data included in this article.

## Author contributions

PD and JS investigated the description of the incident and conceived the study and drafted the manuscript. PD, JS, ZY, and TZ supervised data collection. PD, JS, BK, BZ, and XJ take responsibility for the paper as a whole. PD, JS, ZY, TZ, ZW, TJ, LG, AG, BK, BZ, and XJ contributed substantially to its revision. All authors contributed to the article and approved the submitted version.

## Conflict of interest

The authors declare that the research was conducted in the absence of any commercial or financial relationships that could be construed as a potential conflict of interest.

## Publisher’s note

All claims expressed in this article are solely those of the authors and do not necessarily represent those of their affiliated organizations, or those of the publisher, the editors and the reviewers. Any product that may be evaluated in this article, or claim that may be made by its manufacturer, is not guaranteed or endorsed by the publisher.
